# Community-Based Interventions to Decrease Obesity and Tobacco Exposure and Reduce Health Care Costs: Outcome Estimates From Communities Putting Prevention to Work for 2010–2020

**DOI:** 10.5888/pcd13.150272

**Published:** 2016-04-07

**Authors:** Robin Soler, Diane Orenstein, Amanda Honeycutt, Christina Bradley, Justin Trogdon, Charlotte K. Kent, Kristina Wile, Anne Haddix, Dara O’Neil, Rebecca Bunnell, Communities Putting Prevention to Work Leadership Team

**Affiliations:** Author Affiliations: Diane R. Orenstein, Charlotte K. Kent, Anne Haddix, Rebecca Bunnell, Centers for Disease Control and Prevention, Atlanta, Georgia; Amanda Honeycutt, Christina Bradley, RTI International, Research Triangle Park, North Carolina; Justin Trogdon, University of North Carolina at Chapel Hill, Chapel Hill, North Carolina; Kristina Wile, Systems Thinking Collaborative, Bedford, Massachusetts; Dara O’Neil, ICF International, Atlanta, Georgia. The Communities Putting Prevention to Work Leadership Team: Carolyn Brooks, Harvard School of Public Health, Boston, Massachusetts; Nadine Doyle, Elizabeth Reimels, Amy Holmes-Chavez, Suzi Gates, Rebecca Payne, Centers for Disease Control and Prevention, Atlanta, Georgia.

## Abstract

**Introduction:**

In 2010, the Centers for Disease Control and Prevention (CDC) launched Communities Putting Prevention to Work (CPPW), a $485 million program to reduce obesity, tobacco use, and exposure to secondhand smoke. CPPW awardees implemented evidence-based policy, systems, and environmental changes to sustain reductions in chronic disease risk factors. This article describes short-term and potential long-term benefits of the CPPW investment.

**Methods:**

We used a mixed-methods approach to estimate population reach and to simulate the effects of completed CPPW interventions through 2020. Each awardee developed a community action plan. We linked plan objectives to a common set of interventions across awardees and estimated population reach as an early indicator of impact. We used the Prevention Impacts Simulation Model (PRISM), a systems dynamics model of cardiovascular disease prevention, to simulate premature deaths, health care costs, and productivity losses averted from 2010 through 2020 attributable to CPPW.

**Results:**

Awardees completed 73% of their planned objectives. Sustained CPPW improvements may avert 14,000 premature deaths, $2.4 billion (in 2010 dollars) in discounted direct medical costs, and $9.5 billion (in 2010 dollars) in discounted lifetime and annual productivity losses through 2020.

**Conclusion:**

PRISM results suggest that large investments in community preventive interventions, if sustained, could yield cost savings many times greater than the original investment over 10 to 20 years and avert 14,000 premature deaths.

## Introduction

Approximately half of adults in the United States have heart disease, diabetes, or other chronic diseases; treating chronic disease accounts for approximately 85% of annual health care expenditures (1,2). Decreasing the leading preventable risk factors for chronic disease — obesity and tobacco use — could save lives and substantially reduce health care expenditures (3). Interventions that address these risk factors may make healthier living easier and prevent chronic disease (4). Evidence suggests that establishing conditions for better health requires implementing population-level, high-impact strategies, in addition to individual-level interventions (4).

In 2010, the Centers for Disease Control and Prevention (CDC) launched Communities Putting Prevention to Work (CPPW) to reduce illness, death, and the economic burden of chronic diseases (5). Following a competitive application process, CDC allocated $403 million to 28 communities to prevent obesity, 11 to reduce tobacco use or exposure to secondhand smoke, and 11 to address both obesity and tobacco use for a 3-year period. Awardees included 14 large cities, 12 urban areas, 21 small cities and rural counties, and 3 tribal nations in 32 states and the District of Columbia. Approximately 55 million people lived in these communities (5).

Our objective was to estimate short-term (≤3 years) and long-term (>6 years) benefits of the CPPW interventions that were implemented from March 2010 through June 2013. We analyzed data from several sources including 1) project officer monitoring of awardee action plans, 2) reach estimates, and 3) system dynamics modeling. Recent applications of the RE-AIM (reach, effectiveness, adoption, implementation, maintenance) framework for evaluating public health interventions suggest that reach and effectiveness are important factors to consider when assessing community health interventions (6–8).

## Methods

Awardees developed community action plans (CAPs) that documented the specific evidence- and practice-based policy, systems, and environmental (PSE) interventions they planned to implement to 1) decrease obesity by increasing physical activity and improving nutrition, 2) decrease tobacco use, or 3) decrease exposure to secondhand smoke (5). Interventions were measurable PSE changes, or enhanced implementation of existing or new PSE interventions through use of complementary education or communication interventions.

### Reach estimates: short-term impact

Short-term impacts refer to the number of completed objectives and milestones and estimated number of people reached. To develop early indicators of impact, trained CPPW program officers, subject matter experts, and contractors reviewed awardee data on the number of people they estimated reaching for each intervention in CAPs and analyzed awardee and CDC project officer narratives on program accomplishments. Awardees were provided written guidance that included a list of data sources to use. If needed, awardees could contact a member of the CPPW evaluation team for additional assistance. Reach is defined as the estimated number of people in the target population who have increased access to or are protected by an intervention (5). Determining reach included 1) documenting the setting where the intervention was implemented during the funding period, 2) using census data or setting-specific data (eg, school enrollment) to identify the population count for the setting where the intervention was implemented, and 3) aggregating data.

If interventions were implemented in settings or with populations where there was potential overlap, the overlap was estimated and accounted for in the aggregation process. The population count was capped; the maximum reach a community could have for any one intervention was the census population. Reach included only community residents, not visitors and commuters. Awardees provided reach estimates, which the team validated using census, school enrollment, and other local data sources. We asked awardees to verify the reach estimates. If modifications were made, they were compared with US Census Bureau and local data sources used to generate the original estimates. CDC project officers recorded awardee progress on initiation of interventions quarterly and awardees updated reach estimates in their CAP. We aggregated this information to generate final estimates for the intervention and awardee estimates. We did not weight data according to population characteristics (eg, age, race/ethnicity, sex, income, education) or expected exposure to the intervention but instead applied simple aggregation techniques, using an intent-to-treat approach.

### PRISM overview: long-term estimates

To estimate long-term benefits from 2010 through 2020, we used the Prevention Impacts Simulation Model (PRISM), an interactive, system dynamics simulation model of cardiovascular disease (CVD) prevention that estimates health outcomes, including long-term benefits of prevention of premature deaths and medical and productivity costs averted (9–13). It is designed to forecast how various policies (single or combinations) might influence outcomes (illness, death, or costs caused by CVD, for instance) over a 10- to 30-year span (14). Policy strategies are inputs to the model and are represented as “levers,” or broad categories of approaches to change health behaviors, such as the promotion of healthy food and drink choices or hard-hitting counter-advertising for tobacco. These strategies are translated into their expected effects on PRISM. Users can evaluate many scenarios (combining strategies) by adjusting multiple levers up or down within a predetermined range to reflect the implementation of interventions that address CVD risk behaviors. For example, cigarette taxes reflect state and local taxes and range from 0% to 300% of pretax cigarette prices. The algorithms underlying each lever, including their feasible ranges, are based on published evidence or subject matter expert opinion. Several levers affect CVD risk behaviors in different ways. For example, a tobacco policy may affect tobacco use and exposure to secondhand smoke. PRISM calculates the combined impact of the affected risk behaviors on the incidence and prevalence of chronic illnesses, such as diabetes and obesity, and their subsequent effect on CVD, deaths, and costs.

PRISM is a compartmental model, programmed using Vensim software (Ventana Systems, Inc) that represents population subgroups by age, sex, and prior CVD event. PRISM simulates the distribution of the population across disease risk and health outcome compartments at each point in time, rather than simulating individual disease progression over a lifetime (11,12,14,15). Model outcomes have been validated against historical data from 1990 through 2010 on trends in risk factor prevalence and health outcomes, such as the prevalence of obesity, smoking, and CVD events (14). Simulation modeling complements empirical analysis of public health policies by creating a systematic examination of policy effects on population health behaviors and outcomes in a complex social system (10,12,16).

PRISM simulation outcomes reflect the effect of changes in lever levels if sustained through 2020, compared with baseline trends. Baseline lever levels reflect an awardee’s public health environment pre-intervention. In this way, PSE changes can be evaluated against the unobservable counterfactual scenario of baseline trends continuing over time. For the CPPW analysis, baseline refers to projected trends from 2010 through 2020 on the basis of conditions in the awardee public health environments before implementing CPPW (ie, maintaining status quo).

Each awardee has unique features including population demographics, rates of behaviors and illnesses, and existing PSE. To provide more accurate results for specific local communities, we used local data from the US Census Bureau and the Behavioral Risk Factor Surveillance System to create and calibrate 6 versions of PRISM to reflect CVD risk factors and health outcomes for 6 representative US communities. On the basis of cluster analysis results, we assigned each CPPW awardee to the calibrated version that best reflected awardee features not otherwise captured in PRISM (eg, race/ethnicity, poverty rates) (A. Hardee, J. Trogdon, written communication, February 2011). These locally calibrated versions of PRISM were used for all analyses.

### Translating CPPW into PRISM

Before conducting simulations to estimate CPPW benefits, we translated awardee accomplishments into levers, using a framework that mirrors the approaches other researchers have used to evaluate the impacts of PSE changes (6,17). Fifteen levers were relevant for the CPPW analysis (Table 1).

**Table 1:** Estimated Reach of Selected CPPW Interventions to Improve Nutrition or Physical Activity, Implemented by 50 US Awardees Funded March 2010–June 2013

Intervention^a^	No. of Communities	Estimated Reach
Support development of urban design and land use infrastructure policies	23	36,629,300
Support development of neighborhood/district/jurisdiction plans that support bicycling or walking	20	25,737,800
Improve nutritional content of foods through improved policies, guidelines, or standards	28	23,265,600
Support implementation of nutrition wellness policy	28	19,701,400
Enhance access to healthy food retailer or healthier retail food (not through enhanced transportation)	22	17,262,600
Reduce availability of less healthy foods and beverages	19	15,432,900
Support improvements in food procurement policy	23	15,149,300
Change prices of healthier foods and beverages to match those of less healthy foods	21	11,657,000
Support infrastructure changes to support bicycling or walking	20	11,206,900
Reduce sodium intake through purchasing actions, labeling initiatives, and restaurant standards (not menu labeling)	3	8,567,800
Enhance personal safety in areas where people are or could be physically active (does not include Safe Routes to School)	14	7,866,700
Create places for physical activity	21	5,920,200
Enhance usability of SNAP, WIC, and EBT at healthier food retailers	22	5,796,000
Support implementation of Safe Routes to School	19	5,558,300
Post signs for healthy versus less healthy food items	17	5,010,300

Abbreviations: CPPW, Communities Putting Prevention to Work; EBT, Electronic Benefits Program; SNAP, Supplemental Nutrition Assistance Program; WIC, Special Supplemental Nutrition Program for Women, Infants, and Children.

a Interventions that reached the largest numbers of people, or people in the most communities. They represent 15 of the more than 40 types of nutrition and physical activity interventions implemented by CPPW communities from March 2010 through June 2013. Interventions not included in this table have been described elsewhere (5).

We established baseline levels for each lever by reviewing data and literature on the existing PSE environment for physical activity, nutrition, and tobacco for each awardee. We used city, county, and state information from the literature and data sources, such as the American Lung Association State Legislated Actions on Tobacco Issues database and the National Worksite Health Promotion Survey. Each awardee had the opportunity to review and edit baseline levels to more accurately represent the pre-CPPW environment in their communities. Fourteen awardees reviewed and approved the baseline levels; 9 requested a modification to at least 1 baseline level, which we accepted. For awardees that did not request a modification, we used the baseline lever levels established from the literature and data review. The resulting baseline levers were used to generate baseline outcomes that assumed future policies were kept at status quo.

Communities’ implementation of interventions was expected to affect population behaviors and health in specific ways, as reflected in model assumptions about the impact of lever changes on health outcomes. To estimate how much each intervention would move specific levers, the PRISM review team (3 authors and 3 other analysts) read each awardee’s CAP and determined which levers, if any, might be moved by the objectives and milestones described. For example, an awardee’s intent to “increase access to produce through new farmers’ markets” was linked to the “fruit and vegetable access” lever. Complete lists of CPPW interventions are reported elsewhere (5). Three teams of reviewer-dyads translated completed objectives and milestones into lever movement. Each reviewer estimated the impact of an intervention as a combination of “reach” and “intensity” (7,17). *Reach* is the estimated fraction of people in the target population who have increased access to or are protected by an intervention and ranges from 0 to 1. *Intensity* is the degree to which people exposed to an intervention strategy may change their behavior to make healthier choices (ie, the “effect size” or average percentage change in behavior per person reached). For some levers, intensity is measurable, such as milligrams of sodium consumed; for others, intensity is established relative to benchmarked minimum and maximum index values. For example, for fruit and vegetable access, zero intensity indicates that the interventions linked to the lever resulted in no additional access in the community, whereas an intensity of 1 means that fruits and vegetables were accessible in all possible settings in the community. Team members used an ordinal scale (eg, 0, 0.2, 0.4, 0.6, 0.8, 1.0) to quantify the CPPW change in intensity relative to the baseline lever level. We calculated CPPW-related lever movements relative to baseline lever levels as intensity multiplied by reach. For example, a lever with an intensity of 0.2 and a reach of 0.5 was assigned a lever movement of 0.2 × 0.5 = 0.1 above the baseline level.

### Estimating potential CPPW benefits

After translating completed objectives and milestones into PRISM, we used calibrated models to simulate estimates of the potential benefits, relative to baseline trends, of CPPW achievements for each awardee. Simulations provided estimates of premature deaths, medical costs, and productivity costs averted from 2010 through 2020, assuming that CPPW interventions are fully sustained over time. The year 2010 was used to be consistent with the first program year. Premature deaths averted include those from CVD as well as other chronic diseases, such as type 2 diabetes and lung cancer. Productivity costs averted reflect lifetime productivity costs for premature deaths averted from 2010 through 2020 and illness-related productivity costs for 2010 through 2020. We used a human capital approach to value lost labor and household productivity for adults 18 years or older resulting from deaths and illness attributable to CVD and 7 CVD risk factors (obesity, fruit- and vegetable-poor diet, physical inactivity, smoking [ever smoked], diabetes, hypertension, and psychological distress). The present value of medical and productivity costs was calculated by applying a 3% annual discount rate to costs averted in each year after the start of CPPW (ie, from 2010 through 2020). Cost outputs are in 2008 dollars. Medical costs were inflated to 2010 dollars using the medical care component of the consumer price index; productivity costs were inflated using a measure of growth in mean annual earnings from Occupational Employment Statistics (18).

To estimate the cumulative effect of CPPW, we aggregated the 2010 through 2020 estimates across all awardees. For outcomes that were reported on a per capita basis (eg, premature deaths averted per 1,000 persons), we estimated total potential benefit using US Census Bureau forecasts of the population 18 years or older in each year through 2020.

To examine how sensitive model outcomes are to the input values assumed, we conducted sensitivity analyses that involved randomly selecting values for each of 63 model inputs from assumed distributions for each input. We repeated this process 500 times and ran the model for each randomly selected set of inputs, creating a distribution of outcomes from the 500 model runs. In addition, we examined the impact of uncertainty in the CPPW lever movements by generating both pessimistic and optimistic estimates. We ran the model increasing and then ran the model decreasing each awardee’s lever movements by 10% of the full range for each lever. The lower bound of the sensitivity we report is the 97.5th percentile of the sensitivity analysis results from the pessimistic run, whereas the upper bound is the 2.5th percentile of the sensitivity analysis results from the optimistic run. Lever settings for these analyses were constrained to fall between the awardee’s baseline and maximum lever levels.

## Results

By the end of CPPW, awardees completed 73% of their 790 planned objectives (Box). Population size for the awardee geographic areas ranged from 5,000 to more than 10 million. On the basis of reach projections, an estimated 45.2 million Americans had increased access to physical activity opportunities in schools, afterschool programs, early child care settings, workplaces, and other community settings; 40.9 million Americans had increased access to environments with healthy food or beverage options in schools, afterschool programs, early child care settings, workplaces, and other community settings; and 27.4 million Americans had increased protection from harmful secondhand smoke exposure in workplaces, restaurants, bars, schools, multi-unit housing complexes, campuses, parks, and beaches. Tables 1 and 2 report sample interventions implemented by awardees and the estimated reach of these interventions.

**Box. Examples of CPPW awardee objectives**

CPPW awardees developed intervention objectives as part of their Community Action Plans. Objectives were specific to each awardee. All objectives were SMART, meaning they were specific, measurable, achievable, realistic, and time-limited, as the examples demonstrate.

- By March 2012, support the passage of school and/or county policies requiring daily physical education for middle and high school students jurisdiction-wide.
- By March 2012, develop, adopt, or implement healthy food and beverage policies in at least 3 county departments.
- By March 2012, five hospitals will adopt new tobacco-free campus policies.

**Table 2:** Estimated Reach of Selected CPPW Interventions to Improve Access to Tobacco-Free Environments, Implemented by 50 US Awardees Funded March 2010–June 2013

Intervention^a^	No. of Communities	Estimated Reach
Use evidence-based approaches to restrict tobacco use in public places	21	27,415,300
Enforce existing tobacco policies	14	13,628,400
Support increases in tobacco prices	5	11,897,000
Support development and implementation of zoning restrictions (eg, density of outlets selling tobacco)	9	10,150,200
Restrict sale of tobacco to minors	9	5,418,800
Improve approaches to self-service displays and vending	3	3,358,600
Reduce out-of-pocket costs for cessation therapies (eg, vouchers, changes in insurance)	7	2,763,800
Support reduced distribution of free tobacco samples	7	2,427,100
Restrict point-of-purchase tobacco advertising as allowable under federal law	5	2,221,200
Reduce use of brand-name sponsorships by tobacco companies	3	679,700

Abbreviation: CPPW, Communities Putting Prevention to Work.

a Interventions that reached the largest numbers of people, or people in the most communities. They represent 15 of the more than 40 types of nutrition and physical activity interventions implemented by CPPW communities from March 2010 through June 2013. Interventions not included in this table have been described elsewhere (5).

### Long-term estimates

The lever for physical activity in child-care facilities moved the most, increasing an average of 30% above the baseline lever level for 11 awardees. For 8 of the 15 levers potentially affected by CPPW interventions, average CPPW movement was less than 10% above baseline; for the other 7 levers, average CPPW movement was from 10% to 30% above baseline. 

Results from PRISM simulations indicate that if CPPW interventions from 2010 through 2013 are sustained through 2020, more than 14,000 (range, 2,297–51,199) premature deaths are likely to be averted from 2010 through 2020. Estimated premature deaths averted are almost 9,400 (range, 1,299–36,907) for obesity awardees and 4,600 (range, 998–14,292) for tobacco awardees. Findings also suggest discounted medical costs averted of $2.4 billion (range, $435 million–$8.30 billion) for 2010 through 2020 (2010 dollars), with the largest share of averted medical costs for obesity awardees ($1.6 billion). Estimates of potentially averted productivity losses are $9.5 billion for illness and premature deaths averted from 2010 through 2020.

## Discussion

We described short-term outcomes and estimated long-term potential benefits of CPPW from 2010 through 2020. Estimated lever movements were used to simulate impacts of PSE changes on risk behaviors and on reductions in health and economic outcomes. For awardees who focused on obesity, the greatest impact was for interventions to increase physical activity in schools and child-care facilities and promote physical activity in their communities. For awardees who addressed tobacco, impact was greatest for smoking counter-marketing interventions, such as counter-advertising. These interventions (in some cases a single intervention and in some cases multiple interventions designed to achieve a similar goal) target health behavior directly, had moderate impact, and reached large numbers of people (19,20). The potential long-term benefits of CPPW are considerable when aggregated across all people potentially reached. If CPPW interventions are sustained at their 2013 levels through 2020, findings suggest the potential to avert 14,000 premature deaths, $2.4 billion in medical costs, and $9.5 billion in productivity losses. Although our findings suggest a modest impact on averting deaths, the medical cost per death averted ($28,785) suggests that CPPW was a cost-effective program.

The analysis is subject to several limitations. First, PRISM is a broad CVD model that accounts for most, but not all, strategies implemented in CPPW. Second, PRISM accounts for some intervention implementation costs, such as weight-loss program membership, when estimating net medical costs averted. Because CPPW covered many of these intervention implementation costs ($403 million), comparisons of program costs with PRISM simulations of net medical costs averted may understate the potential benefits. However, any induced costs of CPPW, such as higher costs for healthier foods, were not included. Third, we did not account for costs involved in maintaining interventions beyond the end of funding in 2013. If additional investments are required, policy makers should consider them when comparing program costs with the potential medical and productivity costs averted. If one considers that the $403 million CPPW investment was roughly $134 million per year for 2010 through 2012, an annual investment of 95% of $134 million from 2013 through 2020 would be about $1.02 billion; hence total costs, including implementation, would be $1.42 billion. Because estimated medical costs potentially averted exceed $2 billion through 2020, CPPW would still be considered a cost-saving intervention. Fourth, translating programmatic information (eg, performance monitoring or program results) into any simulation model is difficult and quantifying community PSE changes is inherently subjective, requiring many steps to synthesize qualitative and quantitative data into a quantitative summary measure. The process used here is consistent with approaches used by others to estimate the “dose” for community health interventions (6–8). Finally, this analysis focused on the aggregate impact of CPPW and does not address variability in reach and potential health and economic outcomes for specific awardees.

Some study limitations are expected to result in underestimates of the potential benefit, while others result in overestimates. However, sensitivity analysis results provide a wide confidence range for benefits, because they simultaneously addressed uncertainty in model input values and in estimated lever movements. Nonetheless, the lower bound for direct medical costs averted of $435 million exceeds the $403 million investment in CPPW. These results suggest that even using conservative assumptions, initiatives like CPPW can provide a positive return on investment. Many benefits may be realized over even longer periods because improvements in mortality rates may come decades later, particularly for interventions that target children. Thus, these analyses are generally conservative and likely to underestimate potential long-term gains. Following the CPPW program, PRISM was updated and now includes a separate module that calculates quality-adjusted life years. We anticipate providing outcomes generated from this module in future research.

CPPW supported the implementation of PSE improvements aimed at reducing health-related risk behaviors associated with obesity and tobacco use in awardee communities. These improvements are likely to result in both short- and long-term health and economic benefits, some of which may extend into the future. For the CPPW awardees, system dynamics model simulations suggest the potential for more than 14,000 premature deaths averted, $2.4 billion in discounted direct medical costs averted, and $9.5 billion in discounted lifetime and annual productivity losses averted from 2010 through 2020. Although these findings suggest that more programs that support PSE changes should be implemented, additional research is needed to evaluate similar programs and analyze the cost and benefits of sustaining PSE changes.
